# Effects of hormonal treatment on dermatological outcome in transgender people: a multicentric prospective study (ENIGI)

**DOI:** 10.1007/s40618-022-01944-x

**Published:** 2022-11-08

**Authors:** C. Cocchetti, G. Castellini, M. Maggi, A. Romani, L. Vignozzi, Y. Greenman, M. den Heijer, G. T’Sjoen, A. D. Fisher

**Affiliations:** 1 Andrology, Women’s Endocrinology and Gender Incongruence Unit, Florence University Hospital, Florence, Italy; 2 Psychiatric Unit, Department of Health Sciences, University of Florence, Florence, Italy; 3 Department of Experimental and Clinical Biomedical Sciences “Mario Serio”, University of Florence, 50139 Florence, Italy; 4 Institute of Endocrinology and Metabolism, Tel Aviv Sourasky Medical Center, Tel Aviv University, Tel Aviv, Israel; 5 Department of Endocrinology and Center of Expertise on Gender Dysphoria, Amsterdam University Medical Center, VUmc, Amsterdam, The Netherlands; 6 Department of Endocrinology, Center for Sexology and Gender, Ghent University Hospital, Ghent, Belgium; 7 Viale Pieraccini 6, 50100 Florence, Italy

**Keywords:** Dermatological changes, Gender-affirming hormonal treatment, Acne, Body hair, Alopecia, Transgender

## Abstract

**Purpose:**

The aim of our study was to assess dermatological changes in transgender people after the start of gender-affirming hormonal treatment (GAHT) and to investigate whether various hormonal preparations differently affect dermatological changes in trans AFAB (assigned female at birth) people.

**Methods:**

In a multicenter prospective study, 484 participants (193 assigned male at birth/AMAB and 291 AFAB) were evaluated at baseline (T0), 6 (T1) and 12 months (T2) after the start of GAHT. Hair growth was assessed by the Ferriman–Gallwey (FG) score, acne by the Global Acne Grading Scale (GAGS), and alopecia by the Norwood Hamilton (NH) score.

**Results:**

In AFAB people, a significant increase in FG score and NH grade was observed across time, as well as in GAGS score in a subsample of 71 individuals (*p* < 0.001). Testosterone (T) undecanoate and esters showed a higher increase in hair distribution at T2 vs. T1 as compared to T gel (*p* < 0.01). T esters showed a significantly higher impact in GAGS score modifications at T1 and at T2 vs. T0 compared to T gel (*p* = 0.021 and *p* = 0.003, respectively). In trans AMAB people, a significant decrease of FG score was observed across time (*p* < 0.001), although 51.3% of individuals still reported an FG score higher than eight after 12 months.

**Conclusion:**

T treatment increased hair growth, acne and alopecia prevalence in AFAB people, with T undecanoate and esters influencing hair growth more than T gel. Opposite dermatological changes were observed in AMAB people.

## Introduction

Gender-affirming hormonal treatment (GAHT) usually represents the first medical intervention requested by transgender people in order to align their body with their perceived gender. This implies the administration of testosterone (T) in AFAB (assigned female at birth) trans people and estrogens and/or antiandrogens in AMAB (assigned male at birth) trans people, at variable dosages depending on the requested degrees of de-/feminization and/or de-/virilization [1–4]. Among expected body changes, dermatological effects represent one of the most desired but in some cases also feared effects of GAHT. Indeed, both androgens and estrogens affect the skin pilosebaceous unit (PSU), given the expression of their receptors in the sebocytes and hair follicle dermal papilla [5, 6]. Estrogens’ effects on the PSU remain mostly unknown. They may exert their actions through intracellular or cell surface estrogen receptors (ERα and ERβ), playing a role in skin aging, pigmentation, hair growth and sebum production [7]. On the other hand, T plays a key role in regulating hair follicle proliferation and sebum production through the local conversion of dihydrotestosterone (DHT) by 5 α-reductase [8]. In fact, androgens increase sebum production, hair follicle size, diameter of the hair and anagen phase length [8–10]. Androgens’ action on the PSU may be influenced by 5 α-reductase activity, androgen receptor expression and sensitivity, and hair follicle density per unit skin area [10]. Contrastingly, with a paradoxical mechanism that is still poorly understood, androgens induce miniaturization of hair follicles on the scalp, leading to androgenetic alopecia (AGA) [7].

To date, despite the key role exerted by hair in body image perception in transgender individuals [11, 12], limited research has been conducted in this field, especially in trans AMAB people. Giltay and Gooren [13] assessed hair growth and diameter, acne and sebum production in a small sample of trans AMAB and AFAB people during the first year of GAHT. Their results were subsequently confirmed by two other studies conducted on small samples [12, 14]. No data are available regarding differences in dermatological changes with respect to different T regimens.

Therefore, the main aim of the present study was to prospectively assess mid-term dermatological changes in a large group of transgender people after the start of GAHT. Furthermore, we aimed to investigate whether various hormonal preparations differently affect dermatological changes in trans AFAB people.

## Study design and study population

This study was performed within the European Network for the Investigation of Gender Incongruence (ENIGI) study, a multicentric prospective study resulting from the collaboration of four gender clinics (Ghent, Amsterdam, Florence and Oslo), aiming to evaluate the effect of GAHT on several issues, including dermatological changes [15, 16]. Study design and methods have been extensively described elsewhere [15, 16]. In particular, for the present sub-study, data from Amsterdam, Ghent and Florence were selected. Persons were eligible for inclusion in the study if they were at least 18 years old, had a diagnosis of gender dysphoria based on formal classification criteria [17], and were about to start GAHT. Exclusion criteria included previous or current use of any hormonal preparation, illiteracy/mental retardation, absence of at least a 12-month follow-up, and treatments or disorders affecting dermatological aspects (thyroid disorders, Cushing syndrome, cirrhosis, chronic kidney failure, previous use of antiandrogens or anabolic steroids). Written informed consent was obtained from participants according to institutional guidelines.

The final study sample consisted of 484 participants (291 trans AFAB people and 193 trans AMAB people), enrolled before starting GAHT and evaluated prospectively at baseline (T0), 6 months (T1) and 12 months (T2) after GAHT prescription. All participants reported a binary gender identification and requested a standardized GAHT [2, 4].

All trans AFAB people received T treatment to obtain virilization [1]. Since full virilization was requested, T was administered at the same dosages used in hormone replacement treatment of hypogonadal cisgender men (aiming to achieve T values in the normal male range) [1]. More specifically, intramuscular (i.m.) injections of T undecanoate 1000 mg (the second injection repeated after six weeks, then after 12 weeks) or combination of T esters (250 mg/14 days) was prescribed to 142 and 73 participants, respectively, while transdermal T gel (50–60 mg/day) was prescribed to 76 participants.

As requested, treatment of trans AMAB people consisted of both estrogens and antiandrogens, in order to obtain a full feminization and de-masculinization [1]. All patients received cyproterone acetate (50 mg/daily) combined with estradiol valerate (2 to 6 mg/daily) in 97 participants, estradiol patches (50 to 100 mcg/24 h twice weekly) in 85 participants and estradiol hemihydrate gel (2–3 mg/daily) in 11 participants. Despite different hormonal preparations, in trans AMAB people, target estradiol levels were maintained between 100 and 200 pg/ml with T levels < 50 ng/dL [1].

### Main outcome measures

#### Medical history

All participants completed a self-reported questionnaire with questions regarding medical history, smoking status, previous and current hormonal treatment, medications and dermatological history.

#### Physical examination

At baseline and during each visit, patients underwent a physical examination including measurement of height, weight, body mass index (BMI) and dermatological assessment. The degree of hair growth was assessed by expert endocrinologists using the modified Ferriman and Gallwey (FG) scoring system [18–20]. This score is largely validated only in cisgender population and considers the presence and distribution of terminal hair in nine sites (lip, chin, chest, upper back, sacroiliac region, upper abdomen, lower abdomen, arms and medial thigh). Each site is rated from 0 (no growth of terminal hair) to 4 (very dense hair growth), with a total score ranging from 0 to a maximum of 36. Due to frequent hair removal, in trans AMAB people, the score was adjusted by asking participants to describe medium hair density during the last 2 weeks before depilation or waxing.

Occurrence and pattern of AGA was assessed with the Norwood Hamilton (NH) score [21]. This scale contains seven stages describing typical sequences in the development of male pattern baldness, with higher scores indicating more severe hair loss (for details of different grades, see [22]).

A subsample of patients from Ghent and Florence (AFAB *n* = 71, AMAB *n* = 26) underwent acne assessment. Acne evaluation was performed on the face and back using the Global Acne Grading Scale (GAGS) [23]. GAGS evaluates six locations of the face and chest/upper back, rating each region with a score depending on the type of lesion (no lesion = 0, one comedone = 1, one papule = 2, one pustule = 3, one nodule = 4). These grading scores are then multiplied by a specific factor for each location (forehead ×2, right cheek ×2, left cheek ×2, nose ×1, chin ×1, chest and upper back ×3). After that, the sum of local scores provides the global score (ranging from 0 to 52). Acne severity is graded as mild (global score = 1–18), moderate (global score = 19–30), severe (global score = 31–38) and very severe (global score > 39).

### Biochemical determinations

Venous blood samples were obtained at baseline, 6 and 12 months after overnight fasting, independently from the time of hormonal treatment administration. Samples were analyzed at the local laboratory. Biochemical determinations included T, estradiol, luteinizing hormone (LH) and follicle-stimulating hormone (FSH) levels.

In Amsterdam, serum estradiol levels were measured through a competitive immunoassay (Delfia; PerkinElmer, Wallac Oy, Turku, Finland) with an interassay coefficient of variation (CV) of 13% and a lower limit of quantification (LOQ) of 20 pmol/l until July 2014. Thereafter, liquid chromatography tandem mass spectrometry (LC–MS/MS) was used, with an interassay CV of 7% and an LOQ of 20 pmol/l. Until January 2013, serum T was measured using a radioimmunoassay (RIA) (Coat-A-Count, Siemens, Los Angeles, CA; interassay CV 7–20%; LOQ 1 nmol/l). After January 2013, T levels were determined using a competitive immunoassay (Architect; Abbott, Abbott Park, IL) with an interassay CV range of 6% to 16% and an LOQ of 0.1 nmol/l.

In Ghent, until March 2015 estradiol was measured using E170 Modular (E2 Gen II, Roche Diagnostics, Mannheim, Germany). After that, E170 Modular (E2 Gen III, Roche Diagnostics) with an interassay CV of 3.2% and an LOQ of 92 pmol/L was used. T levels were measured using E170 Modular (E2 Gen II) with an interassay CV of 2.6% and an LOQ of 0.4 nmol/l.

In Florence serum estradiol was measured using electrochemiluminescence immunoassay (E801, Roche Diagnostic) with an interassay CV of 3.6% and an LOQ of 91.8 pmol/l. T levels were measured through E801 (Roche Diagnostics) with an interassay CV of 6.8% and an LOQ of 0.416 nmol/l.

### Statistical analyses

Data were analyzed using IBM SPSS 26.0 (SPSS, Chicago, IL, USA). Sample characteristics are presented as percentages with categorical variables, or as mean ± SD for continuous variables. Data were checked for normal distribution by means of the Kolmogorov Smirnov Statistic. For the assessment of between-group differences (trans AFAB vs. trans AMAB people), a *χ*^2^ and an independent measure *t* test were applied for categorical and continuous variables, respectively. Differences between groups were evaluated in a multivariate model (adjusting for the relevant clinical confounders) by means of an analysis of covariance (ANCOVA) with post hoc Bonferroni test. Statistical significance was determined at *p* < 0.05.

To evaluate changes in dermatological outcomes across time, a mixed linear model was applied to the outcome variable, with visit (number of months of GAHT) as the fixed factor and with a random intercept for baseline scores.

## Results

### Sample characteristics

A total of 484 individuals were eligible for inclusion in the analysis (trans AMAB people *n* = 193, trans AFAB people *n* = 291). Particularly, 62.8% (*n* = 304) of the sample was from Amsterdam, whereas 18.8% (*n* = 91) and 18.4% (*n* = 89) from Ghent and Florence, respectively. No significant differences were found among participants with regards to ethnicity. At the time of GAHT prescription, trans AMAB people were significantly older than AFAB ones (31.21 ± 11.84 vs. 26.15 ± 9.01 years, respectively; *t* = −5.333 *p* < 0.001). Table 1 reports the characteristics of the study sample separately for AMAB and AFAB people, along with significant differences between the two groups.

**Table 1:** Baseline characteristics of the enrolled sample, showed for trans AFAB and AMAB people

		AFAB (*n* = 291)		AMAB (*n* = 193)	*p* value
Study center					
Florence *n* (%)		55 (18.9%)		34 (17.6%)	
Ghent *n* (%)		80 (27.5%)		11 (5.7%)	
Amsterdam *n* (%)		156 (53.6%)		148 (76.7%)	
Age (years)		26.15 ± 9.01		31.21 ± 11.84	*p* < 0.001
Current smoker *n* (%)		110 (37.8%)		50 (25.9%)	*p* < 0.01
Weight (Kg)		71.32 ± 17.79		74.53 ± 14.04	*p* < 0.05
Length (cm)		166.09 ± 7.11		177.77 ± 7.84	*p* < 0.001
BMI (Kg/m^2^)		25.79 ± 5.87		23.54 ± 3.84	*p* < 0.001
Type of gender-affirming hormonal treatment	TE	73 (25.1%)	CPA 50 mg	193 (100%)	
	TU	142 (48.8%)			
	TG	76 (26.1%)	EP	85 (44%)	
			EH	11 (5.7%)	
			EV	97 (50.3%)	

For normally distributed values, mean values ± standard deviation are shown, while categorical data are reported as percentages

*EP* estradiol patches, *EH* estradiol hemihydrate, *EV* estradiol valerate, *CPA* cyproterone acetate, *TU* testosterone undecanoate, *TG* testosterone gel, *TE* testosterone esters, *BMI* body mass index

### Dermatological changes in trans AFAB people

T treatment led to a significant increase of FG score across time (5.63, 95% CI 5.17–6.08). In particular, a significant increase of FG score was observed at all time points (all *p* < 0.001). After 6 months of T treatment, more than half of the participants (62.9%) had a score higher than eight (defining hirsutism in cisgender women) [19]. Regarding AGA, a slight, although significant, increase of NH grade was observed across time (0.07, 95% CI 0.04–0.10) with a significant increase at T2 vs. T1 (*t* = −4.66, *p* < 0.001). Only one participant developed a significant balding after 12 months of T treatment (NH grade III).

**Fig. 1:**
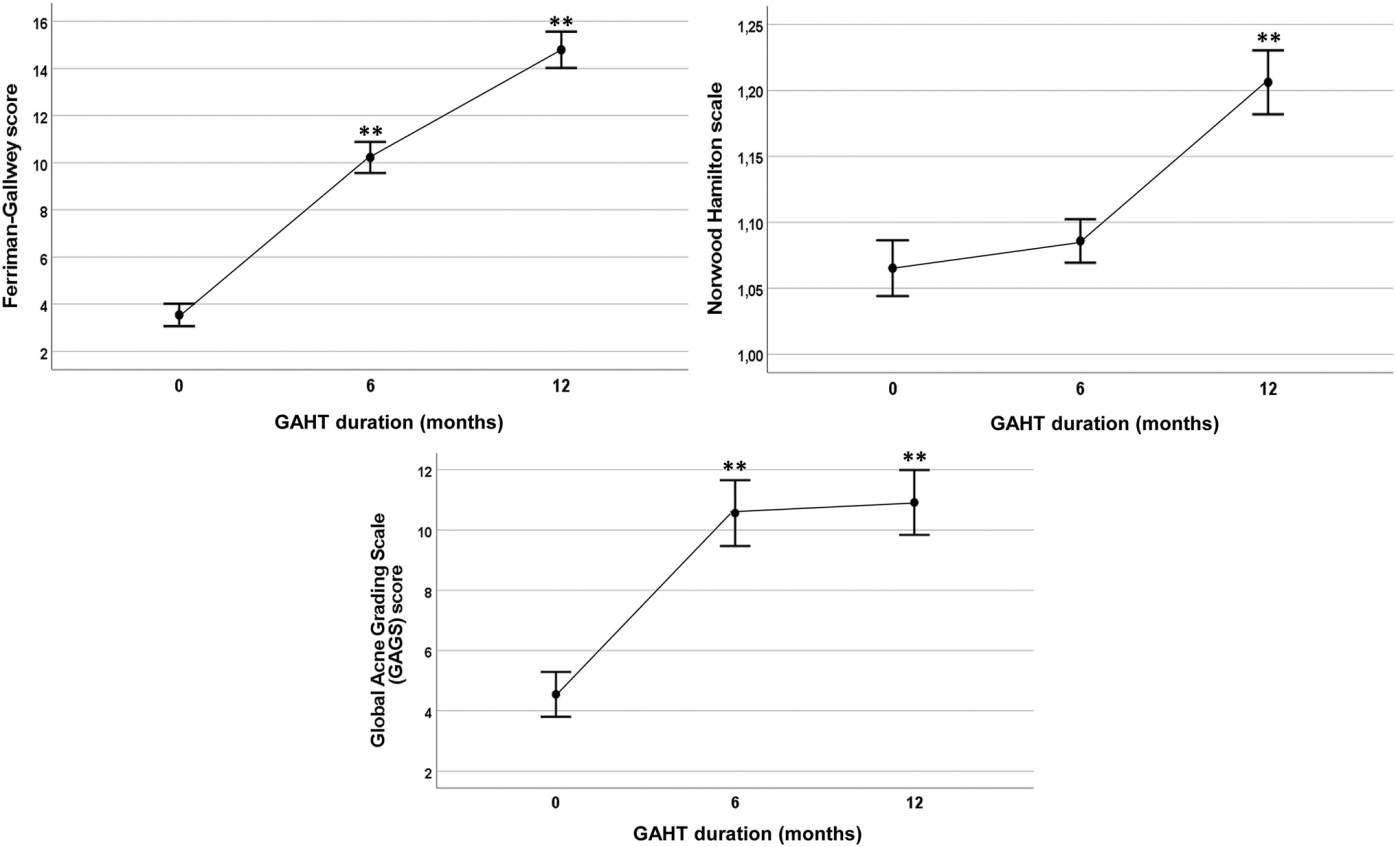
Changes in dermatological outcomes (Ferriman–Gallwey score, Norwood Hamilton score and Global Acne Grading Scale score) in trans AFAB people during gender-affirming hormonal treatment (GAHT). **p < 0.001 across time vs. baseline

In a subsample of 71 individuals, we observed a significant increase of GAGS score during T treatment (2.74, 95% CI 1.45–4.04). In this case, GAGS score showed a significant increase from baseline at T1 (*t* = −6.07, *p* < 0.001), without experiencing a further significant increase from T1 to T2. After six months of T treatment, 4.2% and 18.3% of participants reported, respectively, severe and moderate acne. Figure 1 reports all the aforementioned data.

### Dermatological changes in trans AMAB people

Trans AMAB people reported a significant decrease of FG score across time after the start of anti-androgen plus estrogen treatment (−4.86, 95% CI −5.50 to −4.21). Particularly, FG score showed a marked decrease from baseline to T1 (*t* = 15.02, *p* < 0.001), with a further smaller, but significant, reduction from T1 to T2 (*t* = 8.73, *p* < 0.001). However, after 12 months of treatment the majority of trans AMAB people (51.3%) still reported an FG score higher than eight. Moreover, during the first year of anti-androgen plus estrogen treatment, 64.2% of AMAB individuals underwent cosmetic treatments (i.e., laser removal or electrolysis).

A downward trend, although not significant, was observed across time with regards to NH score. Nevertheless, when adopting paired-sample *t* test to assess differences from one time point to another, a significant reduction was found from T1 to T2 (*t* = 5.76, *p* < 0.001) and from T0 to T2 (*t* = 4.66, *p* < 0.001).

In a subsample of 26 individuals, a significant decrease of GAGS score was found across time (−2.53, 95% CI −4.12 to −0.93). GAGS score showed a significant decrease from baseline to T1 (*t* = 2.82, *p* < 0.01) with no further significant decrease from T1 to T2.

**Fig. 2:**
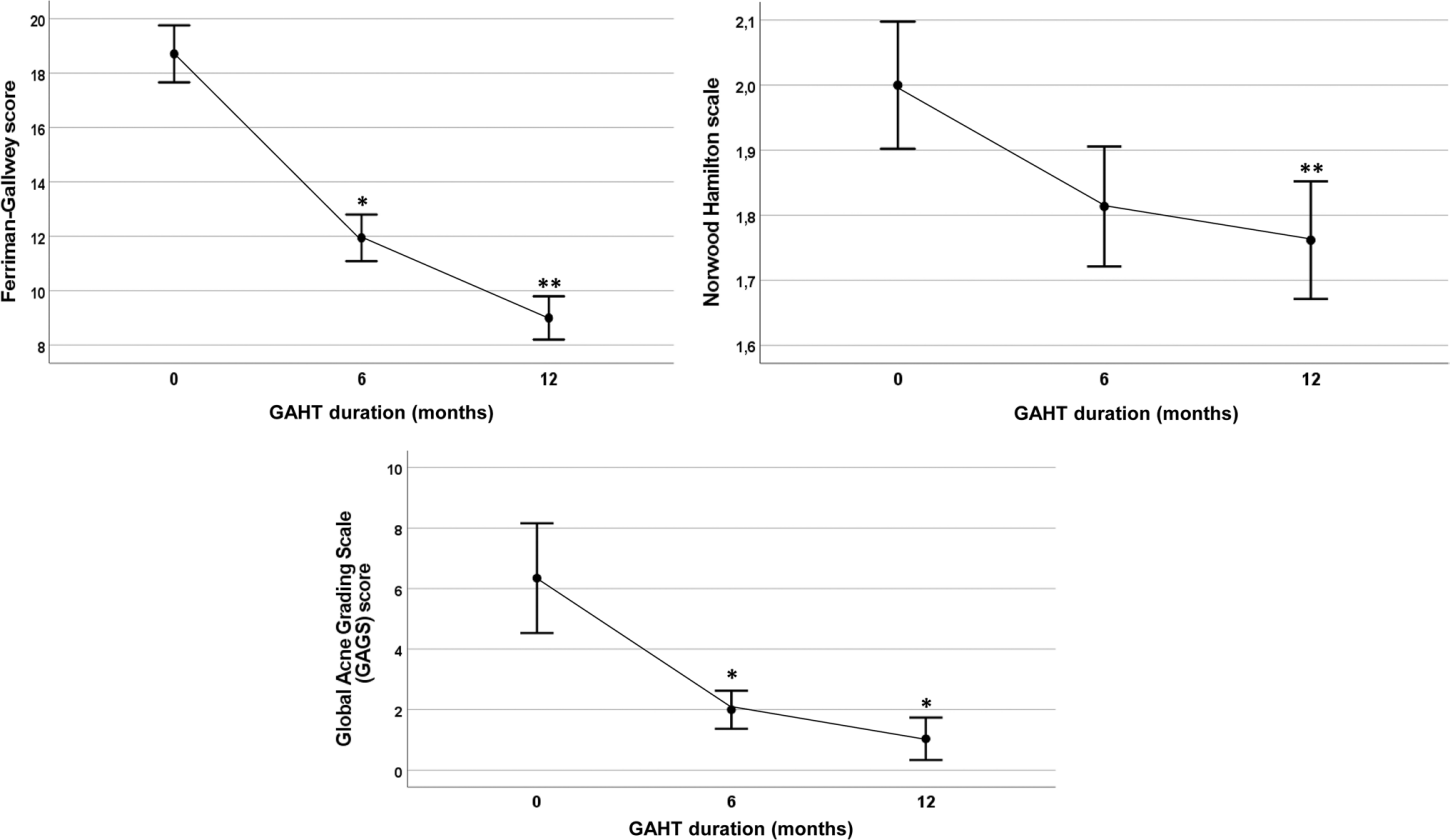
Changes in dermatological outcomes (Ferriman–Gallwey score, Norwood Hamilton score and Global Acne Grading Scale score) in trans AMAB people during gender-affirming hormonal treatment (GAHT). *p < 0.01 across time vs. baseline. **p < 0.001 across time vs. baseline

Figure 2 shows dermatological changes in trans AMAB people.

### Influence of hormonal preparation in AFAB individuals

After excluding from the analysis 38 trans AFAB people who changed T preparation during the first year of treatment, we evaluated differences in terms of dermatological changes with regards to different T preparations (reported in Table 2). Individuals receiving T gel had a higher FG score at baseline compared to those receiving T undecanoate (*F* = 6.71, *p* < 0.001). After adjusting for baseline FG score, increase in hair distribution at T2 vs. T1 resulted as being significantly higher in trans AFAB people treated with T undecanoate and T esters as compared to those using T gel (*F* = 5.26, *p* = 0.006). No significant differences were observed in the rate of change of FG score at T1 vs. T0 among different T preparations. Regarding acne, T esters showed a significantly higher impact in GAGS score modifications at T1 and at T2 vs. T0 compared to T gel (*F* = 4.07, *p* = 0.021 and *F* = 6.45, *p* = 0.003, respectively).

**Table 2:** Comparative analysis on Ferriman Gallwey (FG) and Global Acne Grading Scale (GAGS) scores in trans AFAB people on different T regimens

	Baseline	6 months	12 months
FG score			
TU	1.0 [0–4.25]***	9.0 [5.0–12.0]	14.0 [9.0–18.0]
TE	2.0 [1.0–6.0]	11.0 [7.50–14.50]**	16.0 [11.0–21.0]
TG	4.0[1.0–9.0]	11.0 [9.0–13.0]**	14.0[10.75–18.25]
GAGS score			
TU	2.5 [0–7.0]	8.5 [3.75–15.25]	10 [3.0–15.25]
TE	2.0 ± 3.46	20.0 ± 6.25*	20.67 ± 9.29**
TG	1.5 [0–7.50]	8.5 [2.25–21.50]	10.50 [3.0–30.50]

*TU* testosterone undecanoate, *TG* testosterone gel, *TE* testosterone esters

*p < 0.05

**p < 0.01

***p < 0.001 differences between various hormonal preparations at each time point

### Biochemical measurements

Biochemical changes during GAHT are reported in Table 3. No significant association was observed between changes in FG score, male pattern baldness and GAGS score and T, E2, or LH levels (data not shown).

**Table 3:** Prospective changes of dermatological outcomes and biochemical parameters (testosterone, estradiol, LH, FSH) during gender-affirming hormonal treatment

	Baseline	6 months	12 months	Comparison over time
Trans AFAB people				
FG score	3.54 ± 4.14	10.23 ± 5.70	14.79 ± 6.69	*p* < 0.001
Norwood Hamilton score	1.07 ± 0.36	1.09 ± 0.28	1.21 ± 0.41	*p* < 0.001
GAGS score	1 [0–7]	9 [4–16]	10 [1–16]	*p* < 0.001
Testosterone (nmol/l)	1.2 [0.90–1.60]	20.0 [13.80–28.0]	22.0 [16.0–29.0]	*p* < 0.001
Estradiol (pmol/l)	160.40 [91.8–392.0]	149.80 [113.80–212.0]	154.80 [114.0–199.45]	*p* < 0.001
LH (U/l)	5.1 [2.98–8.33]	3.05 [1.06–7.25]	2.1 [0.29–4.8]	*p* < 0.001
FSH (U/l)	5.9 [3.9–7.6]	4.7[2.7–6.5]	4.8[2.05–6.85]	NS
Trans AMAB people				
FG score	18.66 ± 7.37	11.94 ± 6.03	8.98 ± 5.61	*p* < 0.001
Norwood Hamilton score	2.00 ± 1.36	1.81 ± 1.28	1.76 ± 1.26	NS
GAGS score	1 [0–11]	0 [0–4]	0 [0–0]	*p* = 0.002
Testosterone (nmol/l)	19.0 [14.55–23.50]	0.70 [0.50–0.90]	0.70 [0.50–0.95]	*p* < 0.001
Estradiol (pmol/l)	89.0 [72.0–115.0]	201.50 [138.60–314.25]	200.0 [142.25–332.0]	*p* < 0.001
LH (U/l)	3.4 [2.50–4.50]	0.10 [0.10–0.24]	0.1 [0.1–0.1]	*p* < 0.001
FSH (U/l)	3.87 [2.75–5.28]	0.2 [0.2–0.34]	0.21 [0.1–0.45]	*p* < 0.001

Data are presented as mean ± SD or median (first to third quartiles) in cases of non-Gaussian distribution

GAGS score was evaluated in a subsample (AFAB *n* = 71; AMAB *n* = 26)

*AFAB* assigned female at birth, *AMAB* assigned male at birth, *FG* Ferriman Gallwey, *GAGS* global acne severity score, *LH* luteinizing hormone, *FSH* follicle-stimulating hormone

## Discussion

To the best of our knowledge, this is the first study evaluating the impact of different gender-affirming hormonal preparations on several dermatological aspects in a large multicenter sample of trans AMAB and AFAB people. The main results of the present study are the following: (i) all T preparations resulted as being effective in rapidly increasing hair distribution in trans AFAB people, with i.m. injections of T esters or T undecanoate showing the best outcome; (ii) AGA development was only slightly increased in the first 12 months of T treatment in trans AFAB people; (iii) severity of acne increased during the first year of T treatment peaking after six months, with the higher impact resulting from T esters treatment.

In line with previous reports [12–14], we observed a significant increase of FG score during the first year of T treatment, with a greater effect in the first six months. Indeed, FG score increased progressively from a mean value of 4 at baseline to a value of 15 after 12 months. At 6 months of T treatment, the majority of individuals reported a score indicative of hirsutism in cisgender women [19]. Even if we did not assess satisfaction with hair growth, these results seemed to confirm the efficacy and rapidity of T treatment in inducing the desired dermatological changes in trans AFAB people. When evaluating differences among T regimens, trans AFAB people receiving T undecanoate and T esters had a significantly larger change in mean FG scores than T gel. This may be the consequence of the shorter half-life of T gel, compared to T esters and undecanoate, which may affect its impact on the PSU [24].

Regarding dermatological side effects of T treatment, we found a worsening of hair loss in trans AFAB people during the first year of T treatment. However, the risk of developing significant AGA was low during this period. This result is in line with previous findings, reporting a low prevalence of AGA during the first year of T treatment with a relevant increase after 10 years [14]. In fact, it is likely that the duration of T exposure represents a critical factor for the onset of AGA. However, the prevalence of moderate or severe androgenetic alopecia after a mean T treatment period of 10 years in trans AFAB people is lower compared to the general population [25]. Among possible explanations for this observation, duration of T administration, age at the start of T treatment and differences in PSU’s local T and estradiol ratios have been hypothesized [14].

Acne development represents one of the most common adverse effects of T treatment in trans AFAB people [26, 27]. Similar to previous observations [13, 14], it was found that the presence and severity of acne peaked at 6 months after the start of T treatment. The lack of a further increase between 6 and 12 months could be explained by the start of a specific acne treatment, which was not specifically assessed in our study. Alternatively, it is possible that the initial increase in T levels in trans AFAB people induces sebum production, which then attenuates over time. This hypothesis would be confirmed by the observation of lower rates of moderate/severe acne in the long-term [14]. Furthermore, T esters showed a higher impact on acne development compared to other T regimens. This may be explained by the more pronounced fluctuations of T levels induced by this formulation, reaching supra-physiological levels soon after injection [24].

Literature mostly focused on dermatological changes during GAHT in trans AFAB people and data on trans AMAB individuals remains scarce [12]. Skin PSU is strongly inhibited by anti-androgen plus estrogen treatment in both hair growth and sebum production. Our results highlight the ability of GAHT in bringing trans AMAB people closer to the desired hair pattern. However, despite the limitations of our results, the majority of AMAB participants still had an FG score indicative for hirsutism after 12 months of treatment. Moreover, a high percentage of patients still needed to undergo definitive hair removal procedures. This underlines that for those individuals GAHT alone is usually not enough to achieve their desired hair pattern, making necessary other hair removal systems. Moreover, in the short-term a decrease in T levels had a favorable effect on acne. This subsequently stabilized, probably due to the influence of other mechanisms involved in acne pathogenesis, such as inflammatory pathways.

As expected, no significant associations were found between dermatological changes and sex steroids levels. This may be explained by the differences among T and estrogen preparations and variable timing of sampling with respect to administration. Apart from this, factors other than T levels may affect PSU androgen sensibility, such as genetic differences in androgen receptor sensitivity and/or 5α reductase activity.

## Limitations and conclusion

Our results should be considered in light of some limitations. First, all measures used in our study to assess dermatological changes have a subjective nature. In order to reduce interobserver variability, clinical evaluations were performed by one expert endocrinologist for each center, minimizing the number of examiners. Furthermore, FG method does not consider some androgen sensitive skin areas, such as sideburns and buttocks. However, this scoring system represents the gold standard for evaluation of hirsutism and allows hair growth to be assessed globally. Moreover, hair distribution and density in trans AMAB people were not fully objectively evaluated due to the use of hair removal and cosmetic treatments (i.e., laser removal and electrolysis). Finally, information regarding dermatological therapies for acne and alopecia were lacking.

To conclude, our study has shown that T treatment increased hair growth, acne development and AGA worsening in trans AFAB people during a 12-month follow-up. T undecanoate seemed to influence hair growth more than other T regimens. Antiandrogen plus estrogen treatment induces dermatological changes in the opposite direction in trans AMAB people, even if the latter are usually not sufficient enough to achieve the desired hair distribution.
